# CXCL8 and the peritoneal metastasis of ovarian and gastric cancer

**DOI:** 10.3389/fimmu.2023.1159061

**Published:** 2023-06-12

**Authors:** Xuanrong Fu, Qimeng Wang, Hang Du, Huifang Hao

**Affiliations:** School of Life Sciences, Inner Mongolia University, Hohhot, China

**Keywords:** CXCL8, CXCR1/2, peritoneal, peritoneal metastases, ovarian and gastric cancer peritoneal metastases

## Abstract

CXCL8 is the most representative chemokine produced autocrine or paracrine by tumor cells, endothelial cells and lymphocytes. It can play a key role in normal tissues and tumors by activating PI3K-Akt, PLC, JAK-STAT, and other signaling pathways after combining with CXCR1/2. The incidence of peritoneal metastasis in ovarian and gastric cancer is extremely high. The structure of the peritoneum and various peritoneal-related cells supports the peritoneal metastasis of cancers, which readily produces a poor prognosis, low 5-year survival rate, and the death of patients. Studies show that CXCL8 is excessively secreted in a variety of cancers. Thus, this paper will further elaborate on the mechanism of CXCL8 and the peritoneal metastasis of ovarian and gastric cancer to provide a theoretical basis for the proposal of new methods for the prevention, diagnosis, and treatment of cancer peritoneal metastasis.

## 1 Introduction

Cancer is one of the leading causes of death in the global population. In 2020, globally, there were 19,292,789 cancer cases and 9,958,133 cancer deaths (1). Cancer cases and deaths in China have increased year by year since 2000 (2). Statistics show that in 2022, the top five cancers diagnosed in China are lung cancer, colorectal cancer, stomach cancer, liver cancer, and breast cancer (3). Among these, gastric cancer and colorectal cancer are the third and fifth leading causes of cancer death in China, respectively, and breast cancer is the most common cancer among women (3).

The incidence of peritoneal metastasis is extremely high in ovarian and gastric cancer, while in other cancers (including breast and colorectal cancer) is relatively low, which is shown in **Table 1** (4–9). Classical peritoneal metastasis can be divided into tumor cell shedding, migration, adhesion, invasion, angiogenesis, and other processes. The patients with peritoneal metastastic cancer often experience symptoms, such as nausea, vomiting, abdominal pain, refractory ascites, and adhesive intestinal obstruction. There is currently a lack of effective means for cancers with peritoneal metastases, which leads to the short-term death of patients (10).

**Table 1:** The incidence of peritoneal metastasis in several cancers.

Type of cancer	Incidence of peritoneal metastases	Reference
Ovarian cancer/Epithelial ovarian cancer	62%/70%-75%	(4)/(5)
Gastric cancer	53%-66%	(6, 7)
Breast cancer	7.6%	(8)
Colorectal cancer	5%-15%	(9)

Tumor microenvironment (TME) provides favorable support for tumor growth and life, including tumor cells, endothelial cells, adipocytes, lymphocytes, dendritic cells, chemokines and cytokines (11). Tumor cells interact with surrounding cells through the circulation and lymphatic system to promote their autocrine or paracrine growth factors and inflammatory factors, making the surrounding environment more conducive to tumor development (12). On the other hand, TME can promote immune suppression and immune escape, then further promote the survival and proliferation of tumor cells. Studies show that the occurrence and development of various cancers are closely related to inflammation, and inflammatory factors and immunosuppression in TME play an important regulatory role (13).

CXCL8 is an important pro-cancer inflammatory factor in TME which overexpressed in gastric cancer, ovarian cancer, colorectal cancer, breast cancer, and prostate cancer (14–19). CXCL8 was first discovered to be secreted by monocytes and macrophages and triggers chemotactic eosinophils and T lymphocytes (20). CXCL8 activates multiple downstream signaling pathways by binding to the cell membrane surface receptor CXCR1/2 of various cells (tumor cells, T cells, mast cells), which promotes tumor cell proliferation, migration, invasion, epithelial-mesenchymal transition (EMT) and angiogenesis, further leading to tumor progression (21–24).

After CXCL8 binds to its receptor CXCR1/2, it can directly act on vascular endothelial cells or enhance the activity of matrix metalloproteinase-2 (MMP-2) and matrix metalloproteinase-9 (MMP-9), promote the secretion of vascular endothelial growth factor (VEGF), induce the formation of new blood vessels and increase vascular permeability, which further provides nutritional support for the proliferation and migration of tumor cells (25). Taking CXCL8 and cancer peritoneal metastasis as the starting point, we aim to explain the important role of CXCL8 in ovarian and gastric cancer peritoneal metastasis and provide a theoretical basis for the proposal of new methods for the prevention, diagnosis, and treatment of cancer peritoneal metastasis.

## 2 CXCL8

### 2.1 CXCL8

CXCL8, also known as Interleukin-8 (IL-8), belongs to typical glutamic acid-leucine-arginine (ELR)+CXC chemokines, which can promote angiogenesis (26, 27). The gene encoding CXCL8 is located on chromosome 4q13.3 and consists of 4 exons and 3 introns (28). CXCL8 is a small soluble peptide with a molecular weight of 8-10 kDa. A precursor protein of CXCL8 with 99 amino acids is generated first. Then, a variety of active CXCL8 subtypes form after differential cleavage, including 79, 77, 72, 71, and 69 amino acids (29).

CXCL8 is a multicellular chemokine and is generally produced by monocytes, macrophages, neutrophils, lymphocytes, vascular endothelial cells, and various tumor cells (30–35). CXCL8 recruits neutrophils and other immune cells to inflammatory regions. The aberrant regulation of the CXCL8 pathway has been implicated in many inflammatory mediated diseases, including inflammatory bowel diseases, rheumatoid arthritis, psoriasis, asthma, and cystic fibrosis (36–39). Moreover, it is involved in the occurrence and development of various cancers (40–42).

### 2.2 CXCR1 and CXCR2

**Figure 1:**
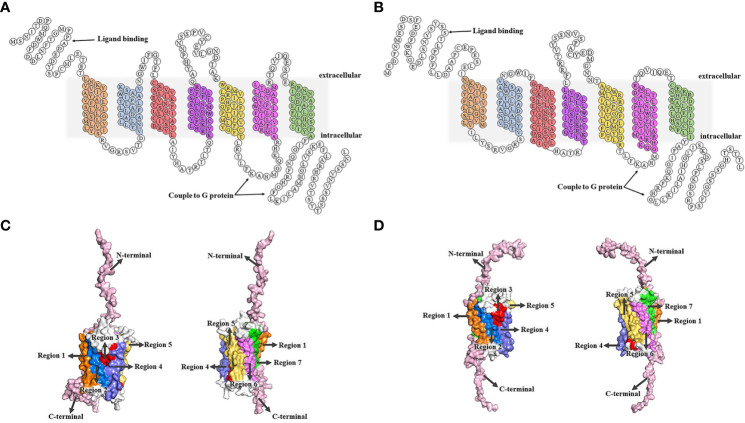
Structures of CXCR1/2. **(A, B)** The 2D structural domains of CXCR1 and CXCR2. The N-terminal of CXCR1/2 is extracellular, while the C-terminal is intracellular. CXCR1/2 contains 7 transmembrane domains, which shown in different colors. The C-terminal and the third intracellular loop are coupled to the G protein, and the N-terminal is the key region for CXCR1/2 to recognize the ligand. CXCR1 binds to CXCL6 and CXCL8, while CXCR2 interacts with CXCL2, CXCL3, CXCL6, CXCL8, etc. **(C, D)** (51). SWISS MODEL online site was used to model all the amino acids of CXCR1 and CXCR2 first. Then, we built tertiary structure of CXCR1 and CXCR2 based on the above 2D structure using the PyMOL Molecular Graphics System. C. Transmembrane regions of CXCR1. The seven transmembrane regions are marked by different colors that correspond to the 2D structural domains of CXCR1. The N-terminal and C-terminal are represented in pink, and the 3 extracellular loops and 3 intracellular loops are shown in white. **(D)** Transmembrane regions of CXCR2. The regions are labeled with different colors that correspond to the 2D structure domains of CXCR2, showing the seven transmembrane domains. The N- and C-terminal are shown in pink, and the 6 loops (3 extracellular loops and 3 intracellular loops) are in white.

After binding to two GTP-binding proteins (G protein) coupled receptors (GPCR), CXC chemokine receptor 1 (CXCR1) and CXC chemokine receptor 2 (CXCR2), CXCL8 induces different biological effects (43, 44). CXCR1 and CXCR2, with approximately 77% amino acid sequence homology, are both located on chromosome 2q35 (45). CXCR1 and CXCR2 were identified and cloned by two research groups at nearly the same time (46, 47). There are 7 transmembrane domains in the middle, including 3 extracellular loops and 3 intracellular loops (48). The N-terminal is located outside the cell and the C-terminal is located inside the cell. CXCR1 consists of 350 amino acids (NCBI Genbank NP_000625.1), and its N-terminal identifies and combines with ligands, such as CXCL6 and CXCL8. The C-terminal and the 3 intracellular loops have the ability to couple to G protein. CXCR2 consists of 360 amino acids (NCBI Genbank NP_001161770.1), and the N-terminal is used for ligand binding, such as CXCL2, CXCL3, CXCL6, and CXCL8. The C-terminal and third intracellular loop are coupled to G proteins (49, 50) (**Figure 1**).

CXCR1 and CXCR2 are widely expressed on the surface of leukocytes including neutrophils, monocytes, T cells, mast cells, basophils natural killer cells, etc. (21, 51, 52). They are also expressed on various tumor cells, such as breast cancer, malignant melanoma, pancreatic cancer, colon cancer, prostate cancer, gastric cancer, and epithelial ovarian cancer (49, 53–57).

### 2.3 CXCL8-CXCR1/2 signaling pathways

The 72-amino-acid dimer that forms CXCL8 is the basic structure that recognizes and binds to the N-terminal of CXCR1/2. Several reports have indicated that CXCR1 binds to CXCL8 with greater affinity than CXCR2, and the binding of CXCR1/2 and CXCL8 can allosterically activate downstream signaling pathways through G protein (44, 58).

**Figure 2:**
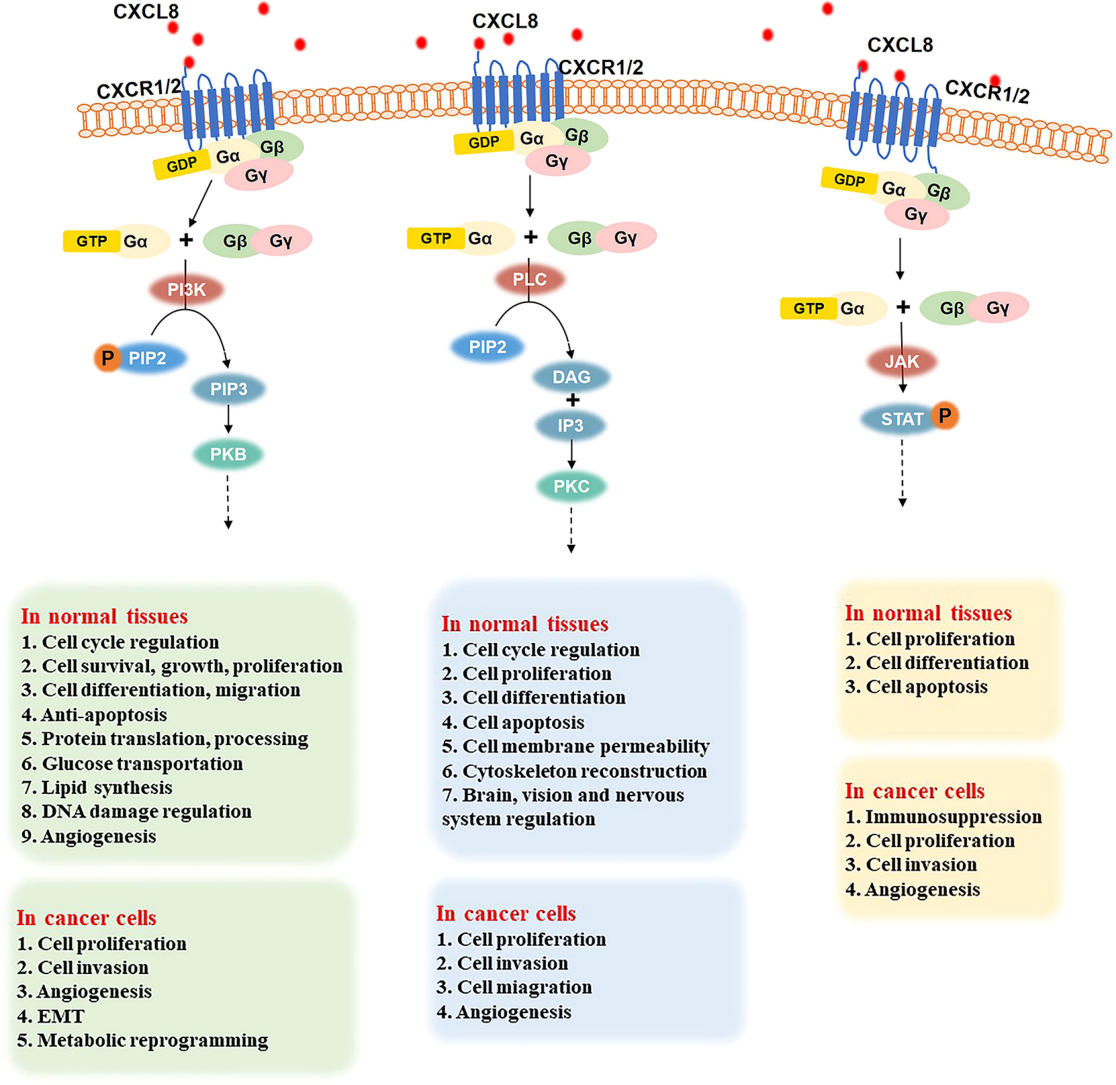
Three important signaling pathways after the binding of CXCL8 to CXCR1/2 and their role in normal tissues and cancer cells. PI3K signal pathway (left): CXCL8 binding to CXCR1/2 activates the G protein, PIP2 is phosphorylated into PIP3 under the effect of PI3K. Then, the combination of PI3K and PKB induces various downstream effectors. In normal tissues, the CXCL8/PI3K/PKB pathway plays a significate role in cell cycle regulation, cell survival, growth, proliferation, differentiation, and migration, anti-apoptosis, protein translation and processing, glucose transportation, lipid synthesis, DNA damage regulation, angiogenesis, etc. In cancer cells, the CXCL8/PI3K/PKB pathway promotes cell proliferation, invasion, metabolic reprogramming, and angiogenesis. PLC signaling pathway (middle): when the CXCL8-CXCR1/2 axis has been triggered, it activates G protein and induces PLC to hydrolyze PIP2 into DAG and IP3. Then, PKC is activated to regulate the cell cycle, cell proliferation, differentiation, apoptosis, cell membrane permeability, cytoskeleton reconstruction, and the brain, vision, and nerve system. In cancer cells, the PLC pathway promotes cell proliferation, migration, invasion, and angiogenesis. JAK-STAT signaling pathway (right): G protein is activated when CXCL8 binds to CXCR1/2. Then, JAK is phosphorylated and activated, further recruiting and phosphorylating STAT. Thus, JAK-STAT pathway regulates cell proliferation, differentiation, and apoptosis in normal cells and improves cell proliferation, invasion, and angiogenesis in various cancers.

G proteins are composed of three subunits: α, β, and γ. In a resting state, the Gα subunit, Gβ subunit, and Gγ subunit form a complex, in which GDP binds to the Gα subunit. The binding of CXCL8 to CXCR1/2 promotes the release of bound GDP by the Gα subunit. Then, GTP binds to the Gα subunit, which results in the dissociation of the Gβ-γ subunits within the complex (27). The GTP-bound Gα and free Gβ-γ subunits initiate signals by interacting with different downstream effector molecules. As shown in **Figure 2**, the following three signaling pathways will be discussed in detail below.

#### 2.3.1 PI3K signaling pathway

After CXCL8 binds to CXCR1/2, G protein is activated. Then, phosphatidylinositol 3 kinase (PI3K) can phosphorylate phosphatidylinositol 4,5-bisphosphate (PIP2) to generate the phosphatidylinositol 3,4,5-triphosphate (PIP3), which is a secondary messenger and mediator of the PI3K pathway (59). Then PI3K binds to protein kinase B (PKB), which contains PH domains in the cell. PKB regulates various downstream effectors through phosphorylation (60). Therefore, the CXCL8/PI3K/PKB pathway plays an important role in cell cycle regulation, cell survival, growth, proliferation, differentiation, migration, anti-apoptosis, protein translation and processing, glucose transportation, lipid synthesis, DNA damage regulation, angiogenesis, and other basic physiological activities (59, 61–65).

In addition, the CXCL8/PI3K/PKB signaling pathway is also over-activated in gastric cancer, ovarian cancer, colorectal cancer, which can promote cell proliferation, metabolic reprogramming, regulate angiogenesis, induce EMT, and improve the infiltration ability of cancer cells (22, 62, 66–70).

#### 2.3.2 PLC signaling pathway

When the CXCL8-CXCR1/2 axis is established, it activates G protein and further induces the phospholipase C (PLC) to hydrolyze PIP2, which is on membrane phospholipids, into two secondary messengers: diacylglycerol (DAG) and inositol-1,4,5-triphosphate (IP3) (71), followed by the activation of the downstream specific protein kinase C (PKC) (71–73). The PLC pathway plays a significant role in many biological activities, such as cell cycle, cell proliferation, cell differentiation (especially bone formation, blood cell, and muscle production), cell apoptosis, cell membrane permeability, and cytoskeleton reconstruction (74–77). Moreover, it is involved in the regulation of infant brain development, vision, and nervous system (78).

Abnormal PLC signaling can be related to brain diseases, such as schizophrenia and Alzheimer’s disease, and cardiovascular diseases, such as cardiac hypertrophy, hypertension, and atherosclerosis (79–81). Various studies have shown that PLC is overexpressed in breast cancer, colon cancer, gastric cancer, hematopoietic malignancies. Therefore, the CXCL8/PLC pathway promotes tumor cell proliferation, migration, and invasion of normal tissues and angiogenesis, which makes cancer worsen and further develop (23, 82–84).

#### 2.3.3 JAK-STAT signaling pathway

G protein activation leads triggers the Janus kinase-signal transducer and activator of transcription (JAK-STAT) signaling pathway when CXCL8 binds to its receptors. STAT stays in the cytoplasm when it is inactive. After encountering the excitation signal, JAK is phosphorylated and activated. Then, JAK recruits and phosphorylates the transcription factor STAT (85), leading to the regulation of the transcription of downstream genes, cell proliferation, cell differentiation, cell apoptosis, etc. (86).

Studies have confirmed that a large number of cytokines and soluble factors send signals through the JAK-STAT signaling pathway to play an important role in inflammation and cancer. For example, these signals affect immunosuppression in prostate cancer, improve the proliferation and invasion ability of cancer cells, enhance angiogenesis in hematopoietic-related malignant tumors, and promote the further development of tumors (24, 86, 87).

## 3 Peritoneal metastasis of ovarian and gastric cancer

### 3.1 Structure and function of the peritoneum

**Figure 3:**
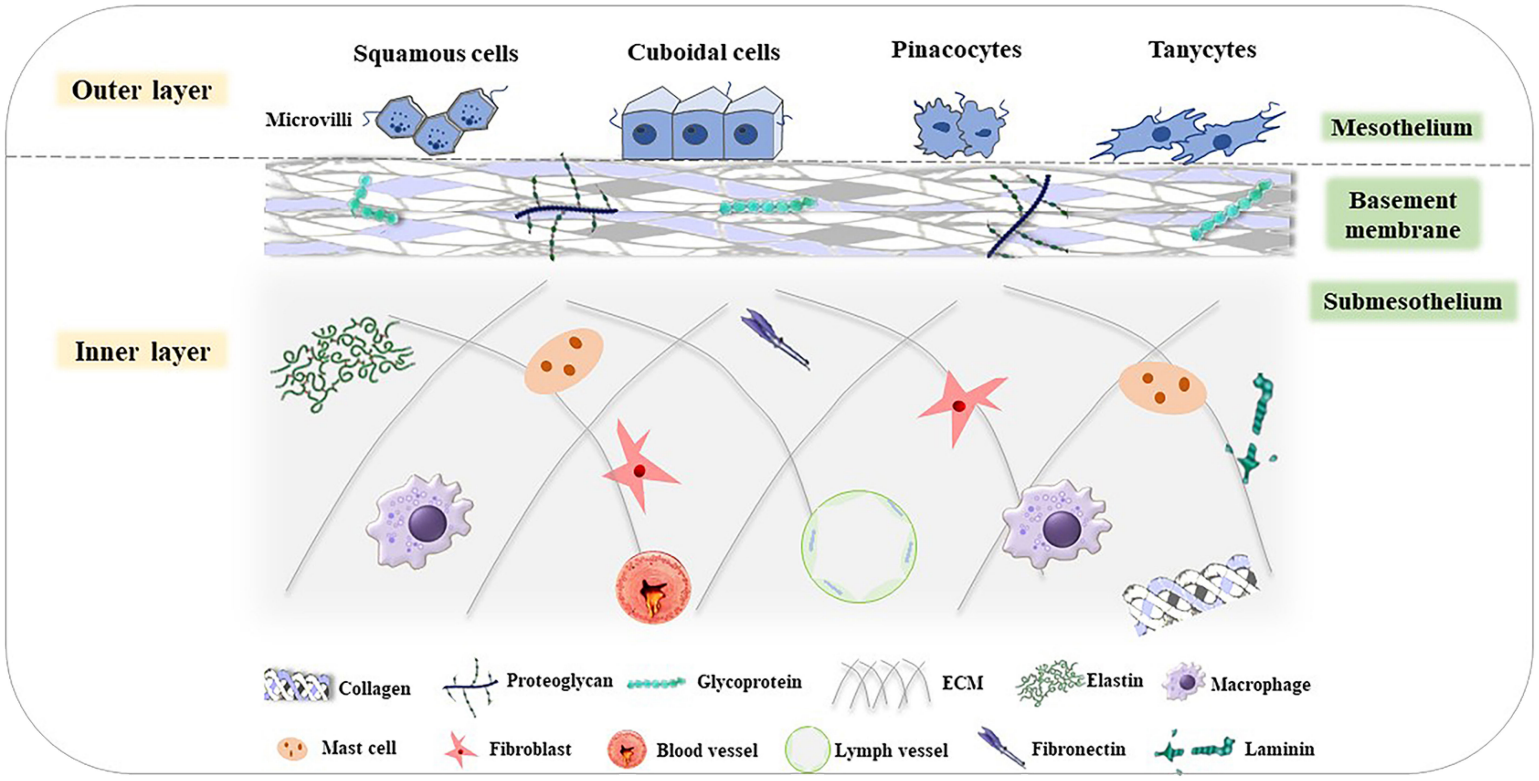
Structure of the peritoneum (85). The peritoneum is divided into the outer layer (mesothelium) and inner layer (basement membrane and submesothelium). In the mesothelium, there are various cells, such as squamous cells, cuboidal cells, pinacocytes, and tanycytes. The basement membrane consists of type I and IV collagen, glycoproteins, and proteoglycans. The submesothelium is composed of ECM, including elastin, and macrophages, mast cells, fibroblasts, fibronectin, blood vessels, lymph vessels, laminin, and so on.

As the surface of the abdominal wall, the peritoneum can be divided into two layers: the inner and outer layer. The outer layer is covered by a monolayer of mesothelial cells with different shapes, such as flat, stretched, squamous-like, and cuboidal mesothelial cells (88). The surface of mesothelial cells has numerous microvilli that have different sizes, shapes, and densities (89). The inner layer is full of tightly arranged cells, which are made up of two parts: the basement membrane and the underlying mesothelial membrane (90). All of these form the niche of peritoneal metastasis. The basement membrane is composed of a thin laminar network and a specialized extracellular matrix (ECM), which contains type I and IV collagen, glycoproteins, and proteoglycans. The underlying mesothelial membrane consists of connective tissues, stromal cells, and ECM. There are some fibroblasts, mast cells, macrophages, blood vessels, and lymphatic vessels in the connective tissues. The ECM includes large amounts of laminin, collagen, fibronectin, and elastin (**Figure 3**) (91–93).

The outer and inner layers of the peritoneum constitute the peritoneal cavity, which has multiple functions: (1) Immobilize the visceral organs and provide them with vascularization and innervation (94); (2) Lubricate both the peritoneum surfaces, which allows for frictionless movements of the viscera, using the interaction of various cells that make up the peritoneal cavity (92, 94); (3) Mediate transmembrane transport. The peritoneum is a semipermeable membrane so that water and dissolved particles can pass between the blood and the peritoneal cavity (92, 95, 96); (4) Promote the inflammation reaction. Peritoneal cells interact with each other by their autocrine or paracrine secretion products to regulate inflammation (92, 97). In this process, peritoneal macrophages (PMs) generate a great amount of tumor necrosis factor-α (TNF-α) first. Then, peritoneal mesothelial cells (PMCs) are also able to secrete a large amount of cytokines (CXCL1, CXCL6) and chemokines (CXCL8), monocyte chemotactic protein 1 (MCP-1), growth factor (transforming growth factor-β (TGF-β), VEGF) and adhesion molecules (E-cadherin) into the inflammatory microenvironment (98–100).

### 3.2 Peritoneum-associated cells

#### 3.2.1 Peritoneal mesothelial cells

PMCs are unique cells with dual mesenchymal-epithelial characteristics that are located on the inner wall of the peritoneal cavity (94). PMCs are typical fibroblasts that originated from the mesoderm. However, their appearance and function are more similar to epithelial cells. Thus, PMCs express the typical protein markers of mesoderm and epithelial cells, including vimentin and keratins, respectively (100, 101).

PMCs have multiple functions: (1) Make up the peritoneum. PMCs are the largest cells among all the components that form the peritoneal cavity; (2) Maintain homeostasis of the intraperitoneal (102); (3) Form a smooth surface to reduce the friction of the internal organs (98, 101); (4) Tissue repair. PMCs can secret various growth factors, including TGF-β, platelet-derived growth factors (PDGF), fibroblast growth factors (FGF), and VEGF, to promote cell proliferation and recovery at the site of injury (103);(5) Inflammation regulation. As metabolically active cells, PMCs secret various mediators to participate in inflammation regulation. It is demonstrated that bacteria attached to the PMCs can be phagocytosed (104), which further activates the PMCs and releases CXCL8 (105). On the other hand, PMCs will generate growth-related oncogene-α(GRO-α)to let T cells secret cytokine IL-17 (106). Other studies have also shown that PMCs secret IL-6, which plays an important role in promoting inflammation (106, 107);

#### 3.2.2 Peritoneal macrophages

PMs are generally concentrated on milky spots,which are actually primitive lymphoid tissue (108). As the entrance to the lymphatic vessels, milky spots are abundant on the surface of the omentum and lower peritoneum. Their primary role within the structure is associated with the absorption and elimination of debris and bacteria from the peritoneum.

Under normal circumstances, an important role of PMs concerns the regulation of the immune reaction within the peritoneal cavity. PMs have the capacity to secret C-C motif chemokine ligand 22 (CCL22), which can recruit immunosuppressive Treg cells into tumors (109). It has also been demonstrated that the CCL22 level in ovarian cancer patients is higher than in benign tumor patients (110).

### 3.3 Formation of ovarian and gastric cancer peritoneal metastasis

Ovarian cancer is the most common cancer-causing death in gynecological malignancies (111). Ovarian cancer cells have the ability to metastasize throughout the peritoneal cavity, including the liver, gallbladder, uterus, spleen, and other parts. However, in addition to the fallopian tube and lateral ovary, the peritoneum and omentum are the most common secondary sites (112), the incidence of peritoneal metastasis in ovarian is about 62% (4). At the same time, that peritoneal metastatic spread is an integral part of FIGO staging of ovarian cancer (113). All cells in the ovary can cause malignant tumors, of which epithelial ovarian cancer (EOC) is the most common and lethal, accounting for about 80% of ovarian cancers (114, 115). About 70%-75% of women patients with epithelial ovarian cancer are found to have cancer cells metastasized to the peritoneal cavity and the five-year survival rate is only 30% (5, 116).

Gastric cancer is a malignant tumor originating from the gastric mucosal epithelium, with the third-highest mortality rate in the world (117). Peritoneal metastasis is common in the recurrence and metastasis of gastric cancer, about 53%-66% (6, 7). Studies have demonstrated that cancer recurrence in about 50% of gastric cancer patients was confined to the peritoneal cavity. Peritoneal metastasis of gastric cancer is often accompanied by symptoms, such as refractory ascites and adhesive intestinal obstruction, which causes patients to die in a short period of time (118).

**Figure 4:**
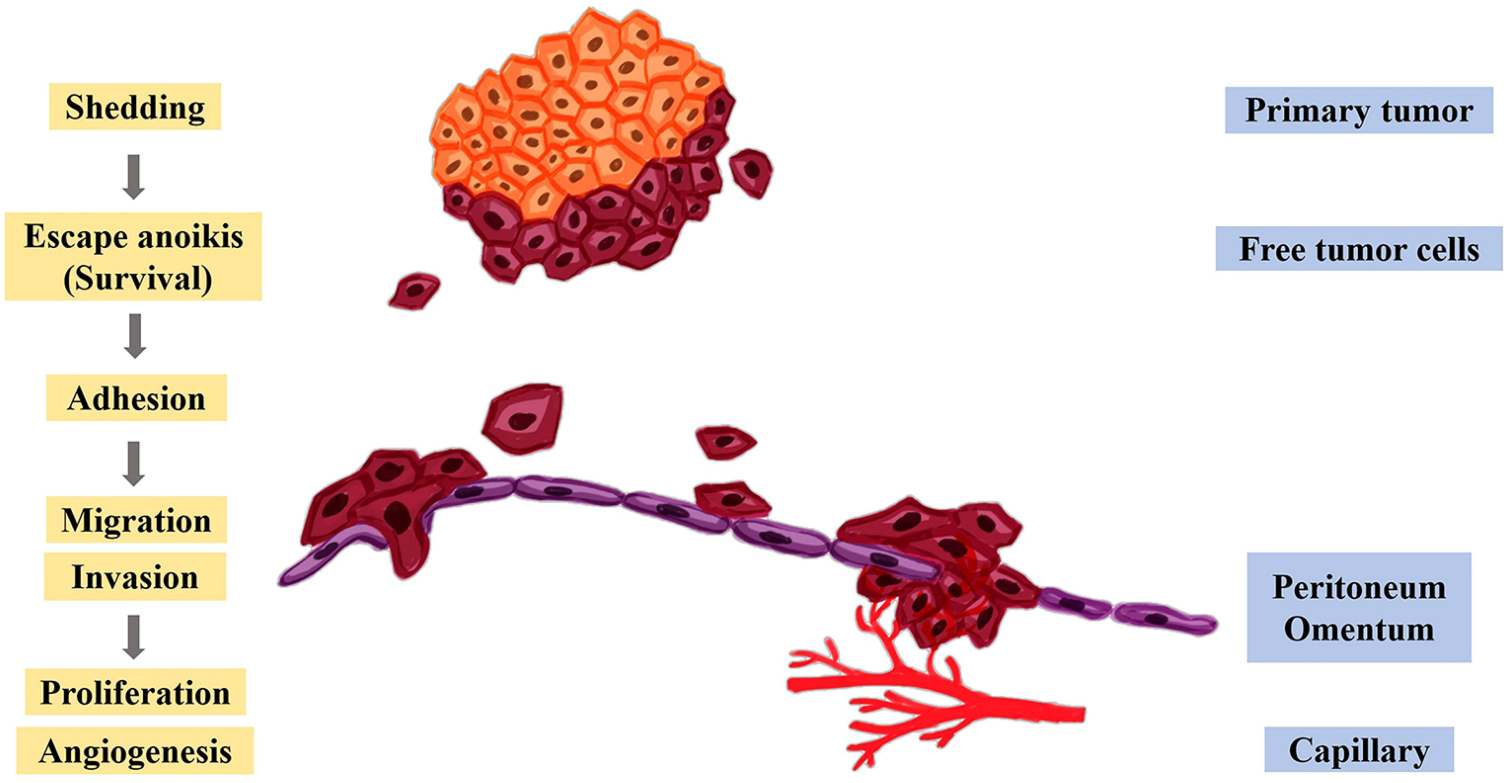
Molecular mechanism of peritoneal metastasis. After the cancer cells fall off the primary tumor, they escape anoikis, survive in the abdominal cavity, and further adhere to the peritoneum and omentum, thereby promoting the proliferation, migration, invasion, and angiogenesis of tumor cells.

The peritoneum has an extensive area, which makes it an excellent site for the development of secondary tumors (114). Yet, when peritoneal fluid, which is in the peritoneal cavity, flows in the cavity, it can gather tumor cells and distribute them to some extent in a stochastic manner throughout the whole cavity (119). Peritoneal metastasis can be described vividly by Stephen Paget’s “seed and soil” theory. Only the cancer cells (the “seeds”) that transfer to a usable environment (the “soil”) can they survive and proliferate rapidly (120). Peritoneal metastasis mainly includes the following processes, as shown in **Figure 4**. The free cancer cells formed by the shedding of the metastatic primary tumor gain motility in the peritoneal cavity and avoid anoikis first. The surviving free cancer cells contact and adhere to the monolayer of mesothelial cells in the peritoneal. Then, the peritoneal deposits are formed on the surface of the peritoneal. Meanwhile, the tumor cells and immune cells within the peritoneal cavity produce and release inflammatory factors, which further changes the structure of the peritoneum, facilitates the proliferation, migration invasion of tumor cells and promotes angiogenesis (91, 121).

(1) Shedding. Metastatic primary ovarian and gastric cancer cells can become free by spontaneous shedding (122, 123). Alternatively, epithelial cells increase their motility to promote spontaneous shedding through EMT, with the upregulation of N-cadherin and downregulation of E-cadherin (124, 125).

(2) Acquisition of anoikis resistance. Free cancer cells are suspended in peritoneal fluid and cannot attach to other cells or the ECM. Thus, avoiding anoikis is a necessary condition for the survival of ovarian and gastric cancer cells (115). Protein-L-isoaspartate (D-aspartate) O-methyltransferase (PCMT), which is localized to various regions, including the cytosol, extracellular space, exosomes, and possibly vesicles (126, 127). PCMT interacts with the carboxymethylation of the tumor suppressor protein p53 at residues 29 and 30 of isoaspartate and downregulates its expression, thereby inhibiting the anoikis of tumor cells and promoting tumor development (128). Studies have shown that knocking out the PCMT gene in ovarian cancer significantly leads to anoikis (129).

In addition, related research shows that PMCs display poor proliferative capacity and fast entry into senescence. Senescent PMCs will secrete senescence-associated secretory phenotype (SASP) into the surrounding environment, which usually includes cytokines, chemokines, growth factors, and matrix-related basic proteases (101). Many biologically active substances make the senescent PMCs more effective than vigorous cells to promote the adhesion of ovarian cancer, colon cancer, and pancreatic cancer cells. At the same time, senescent PMCs can secret some related proteins that have the ability to promote cell proliferation, angiogenesis, inflammation, and EMT, simultaneously improving the motility of cancer cells, and finally further promoting the peritoneal metastasis of cancer (130–133).

(3) Adhesion. Surviving free cancer cells contact and adhere to PMCs. Adhesion can be divided into two mechanisms: transmesothelial and translymphatic. During transmesothelial dissemination, free cancer cells adhere directly to the innermost mesothelial layer of the peritoneum (134). Adhesion among tumor cells, mesothelial monolayers, and ECM components is achieved through interactions between integrins, cadherins, and cell adhesion molecules, including integrin α2β1, proteoglycan CD44, and mucin 16 (MUC16). Several studies have shown that integrin α2β1 and proteoglycan CD44 are overexpressed in ovarian and gastric cancers (135–137). Integrin α2β1 promotes ovarian cancer cells to form spheres and enhances their adhesion ability to facilitate peritoneal metastasis (138). CD44 can enhance cell adhesion and cell migration (139). MUC16 is involved in mediating the adhesion of free tumor cells, which is mainly achieved by interacting with mesothelin on mesothelial cells (140).

(4) Invasion. Adhering tumor cells must cross the mesothelial monolayer to invade the peritoneum. The single cells or spheroids formed after ovarian cancer cells break through the body’s immune defenses and invade the peritoneal stroma (115). In gastric cancer, PMCs can synthesize inflammatory factors and angiogenic factors, such as VEGF, fibroblast growth factors (FGF), etc. (141), which further promotes matrix remodeling and angiogenesis to change the peritoneal structure, destroying the intact mesothelial monolayer and leading to the formation of a large number of matrix deposits in the submesothelial dense area. All of the above are beneficial to the invasion of gastric cancer cells (142).

(5) Proliferation and angiogenesis. One of the typical characteristics of tumor cells is the ability to proliferate indefinitely. Cancer cells produce various growth factors through autocrine activators or stimulate the relevant stromal paracrine factors in TME and integrate with the corresponding receptors to further promote tumor cell survival, proliferation and angiogenesis (143). When the ovarian and gastric cancer cells colonizes the peritoneum and grows to a certain size, VEGF will play a significate role in increasing the formation of new blood vessels by stimulating the proliferation and migration of endothelial cells, enhancing vascular permeability, and inhibiting the apoptosis of endothelial cells to provide the nutrients needed for tumor growth (142, 144). Another role of the PMs is their contribution to angiogenesis, as they produce various proangiogenic stimuli, such as VEGF, (matrix metalloproteinase-1) MMP-1, and so on (145).

## 4 CXCL8-CXCR1/2 axis promotes the peritoneal metastasis of ovarian and gastric cancer

**Figure 5:**
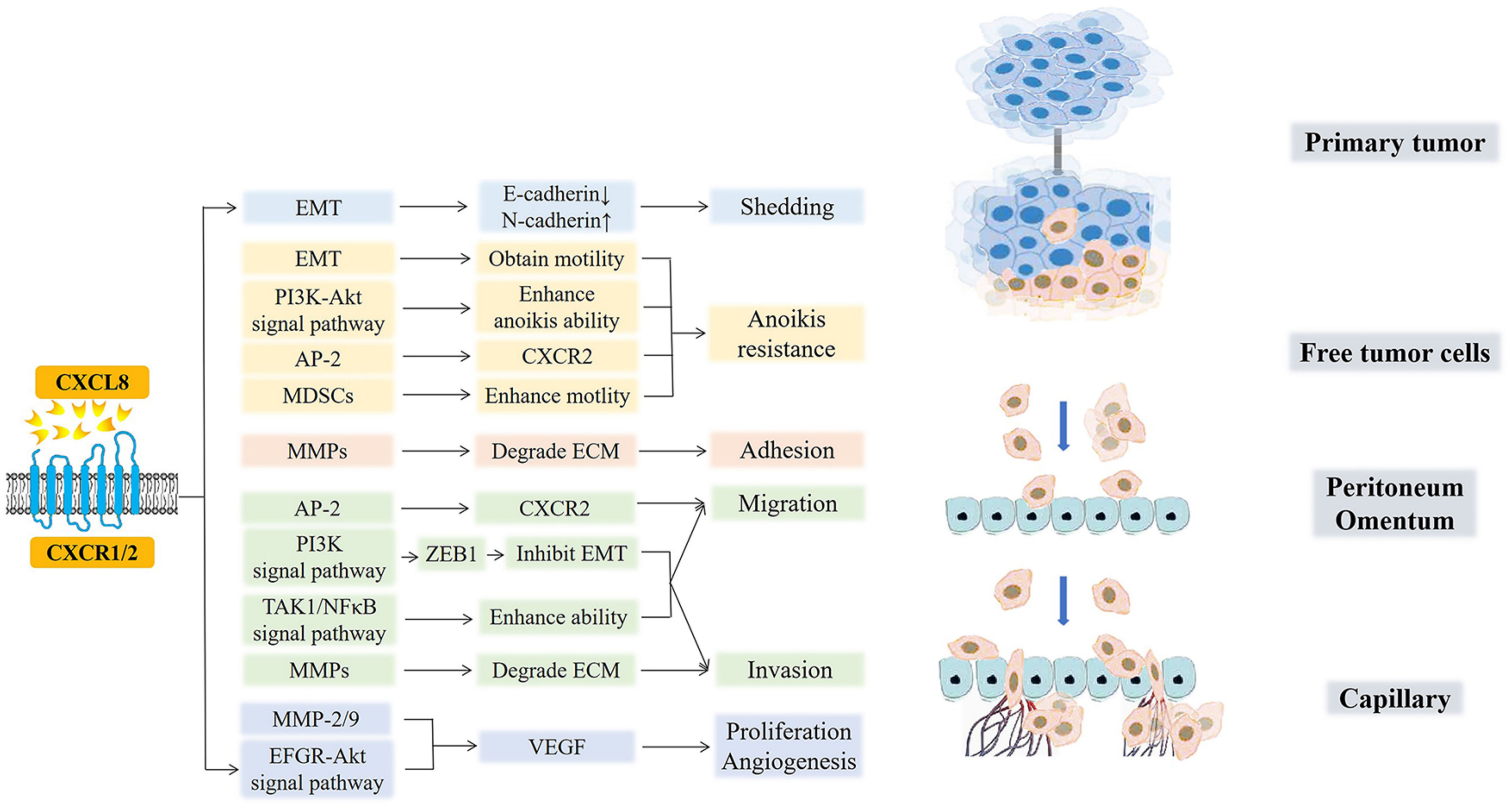
CXCL8 and cancer peritoneal metastasis. CXCL8 promotes the occurrence, development, and peritoneal metastasis of cancers in multiple ways. CXCL8 accelerates tumor cell shedding via EMT (E-cadherin decreased, N-cadherin increased). Moreover, EMT and MDSCs, which are recruited by CXCL8, free tumor cells to enhance motility and to acquire anoikis resistance. AP-2 promotes the hexaphosphorylation of serine residues on CXCR2 and then, improves the migration of tumor cells while avoiding anoikis. After that, the CXCL8-CXCR1/2 axis enhances the anoikis ability by the PI3K-Akt signaling pathway. In the next step, CXCL8 induces MMPs to degrade ECM for the adhesion and invasion of free tumor cells. Then, CXCL8 facilitates the expression of ZEB1 through the PI3K signaling pathway, which directly inhibits EMT, further promoting the migration and invasion of cancer cells. Furthermore, CXCL8 can activate TAK1/NFκB signaling and further enhance the tumor cells’ migration and invasion abilities. Finally, CXCL8 can active the EFGR/Akt signaling pathway and enhance the activity of MMP-2/9 to promote the secretion of VEGF, which further induces the formation of new blood vessels and provides nutritional support for the proliferation and migration of tumor cells.

Paracrine or autocrine CXCL8 in TME can promote the occurrence and development of the peritoneal metastasis of various cancers in multiple ways, as shown in **Figure 5** (146–148).

(1) Shedding. In the process by which tumor cells separate and shed from the primary tumor, it is necessary to adjust the expression of adhesion molecules on the surface of the tumor cells (125). CXCL8 mediates EMT in ovarian and gastric tumor cells by binding to its receptor so that the down-regulation of E-cadherin and up-regulation of N-cadherin reduce the adhesion between tumor cells and induce tumor cell shedding (149). This reversible biological process also enhances the anti-anoikis ability of cells and give the cells more mobility, so the free tumor cells can find the most suitable adhesion position in the peritoneal environment. In all, one of the main ways that ovarian and gratric tumor cells can shed and acquire invasiveness and motility is through EMT (124).

(2) Acquisition of resistance to anoikis. CXCL8 can enhance the anti-anoikis function by activating the PI3K-Akt signaling pathway in ovarian cancer [210]. Further, Adapyin-2(AP-2) can promote the hexaphosphorylation of serine residues on CXCR2, which then improves the migration ability of tumor cells while avoiding anoikis (150). Moreover, CXCL8 recruits myeloid-derived suppressor cells (MDSCs) to TME, which enhances the motility of tumor cells, endothelial cells, or tumor-associated leukocytes and promotes the immune evasion of tumor cells (151–153). Under the action of exogenous chemotherapeutic drugs, excessive CXCL8 can inhibit the apoptosis of ovarian and gastric cells induced by chemotherapeutic drugs and improve the drug resistance of cancer cells (18, 154–156).

(3) Adhesion and invasion. The increase in autocrine or paracrine production of tumor cells with degrading collagen fibers (matrix metalloproteinases, (MMPs)) leads to the contraction and lysis of ECM cells, which facilitates tumor cell adhesion. In addition, CXCL8 can enhance the activity of MMPs and promote the degradation of ECM by MMPs, which is beneficial for the invasion of gastric cancer cells (157). Grb2-associated binding protein 2 (GAB2), a scaffolding protein necessary for ovarian cancer peritoneal metastasis, is overexpressed in cancers. GAB2 can activate CXCL8 and induce the expression of zinc-finger E homeobox-binding-1 (ZEB1) through the activation of the PI3K signaling pathway, which directly inhibits the transcription of E-cadherin, further promoting the migration and invasion of ovarian cancer cells (158, 159). GAB2 protein can also undergo tyrosine phosphorylation after binding to receptors and interact with the p85 regulatory subunit of PI3K containing Src homology 2 domain molecules, improving the survival rate of tumor cells and promoting their proliferation and migration (160). Further, when CXCL8 binds to CXCR1/2, it can activate transforming growth factor beta-activated kinase 1/Nuclear factor-kappa B (TAK1/NFκB) signaling and enhance the migration and invasiveness of ovarian cancer cells. In addition, it can also activate the EFGR/Akt signaling pathway to inhibit apoptosis and angiogenesis (49).

(4) Proliferation and angiogenesis. After CXCL8 binds to the receptor CXCR1/2, it can directly act on vascular endothelial cells or enhance the activity of MMP-2/9, which promotes the secretion of a large amount of VEGF and enhances its activity, inducing the formation of new blood vessels and increasing vascular permeability, further providing nutritional support for the proliferation and migration of ovarian cancer cells (161, 162). At the same time, CXCL8 derived from gastric cancer have the ability to activate endothelial cells in cancer vessels, promote angiogenesis (163). In conclusion, angiogenesis is essential for tumor cell invasion and colonization in mesothelial or stromal cells during peritoneal metastasis (164, 165).

To summarize, the overexpression of CXCL8 promotes tumor cell proliferation, migration, invasion, EMT, and angiogenesis and accelerates peritoneal metastasis. peritoneal-associated cells also upregulate CXCL8 expression. CXCL8 interacts with peritoneal metastases to accelerate tumor progression and leads to a dramatic deterioration of patients.

## 5 Conclusions

Peritoneal metastasis frequently occurs in ovarian and gastric cancer, resulting in poor prognosis and a low five-year survival rate, which is the main reason for patients’ death. This paper details the structure and roles of autocrine or paracrine chemokine CXCL8, which is in the TME, and the membrane receptor CXCR1/2. We further describe the specific process of peritoneal metastasis in ovarian and gastric cancers. Finally, the CXCL8-CXCR1/2 axis greatly affects the promotion of ovarian and gastric cancer peritoneal metastasis, further facilitating the development and progression of cancer. It is expected to indirectly reduce the probability of peritoneal metastasis by developing a series of drugs targeting CXCL8 or CXCR1/2, or by improving the treatment effect of intraperitoneal chemotherapy on patients, slowing the deterioration of cancer after peritoneal metastasis.

## Author contributions

All authors read the manuscript and provided feedback. XF wrote the paper. QW and HD provided the figures. HH revised the manuscript. All authors contributed to the article and approved the submitted version.

## References

[1] Sung H Ferlay J Siegel RL Laversanne M Soerjomataram I Jemal A. Global cancer statistics 2020: GLOBOCAN estimates of incidence and mortality worldwide for 36 cancers in 185 countries. CA Cancer J Clin (2021) 71(3):209–49. doi: 10.3322/caac.21660 33538338

[2] Wei W Zeng H Zheng R Zhang S An L Chen R. Cancer registration in China and its role in can-cer prevention and control. Lancet Oncol (2020) 21(7):e342–9. doi: 10.1016/S1470-2045(20)30073-5 32615118

[3] Xia C Dong X Li H Cao M Sun D He S. Cancer statistics in China and united states, 2022: profiles, trends, and determinants. Chin Med J (Engl) (2022) 135(5):584–90. doi: 10.1097/CM9.0000000000002108 PMC8920425 35143424

[4] Riihimäki M Thomsen H Sundquist K Sundquist J Hemminki K. Clinical landscape of cancer metastases. Cancer Med (2018) 7(11):5534–42. doi: 10.1002/cam4.1697 PMC6246954 30328287

[5] Peng J Yang X Li X Gao H Liu N Guo X. 1-calcium phosphate-uracil inhibits intraperitoneal metastasis by suppressing FAK in epithelial ovarian cancer. Cell Cycle (2019) 18(16):1925–37. doi: 10.1080/15384101.2019.1634946 PMC6681791 31290719

[6] Coccolini F Cotte E Glehen O Lotti M Poiasina E Catena F. Intraperitoneal chemotherapy in advanced gastric cancer. meta-analysis of randomized trials. Eur J Surg Oncol (2014) 40(1):12–26. doi: 10.1016/j.ejso.2013.10.019 24290371

[7] Fujitani K Yang HK Mizusawa J Kim YW Terashima M Han SU. Gastrectomy plus chemotherapy versus chemotherapy alone for advanced gastric cancer with a single non-curable factor (REGATTA): a phase 3, randomised controlled trial. Lancet Oncol (2016) 17(3):309–18. doi: 10.1016/S1470-2045(15)00553-7 26822397

[8] Yu JH Feng Y Li XB Zhang CY Shi F An SL. Cytoreductive surgery and hyperthermic intraperitoneal chemotherapy for peritoneal metastasis from breast cancer: a preliminary report of 4 cases. Gland Surg (2021) 10(4):1315–24. doi: 10.21037/gs-20-893 PMC8102231 33968683

[9] Kim CH. Molecular analyses in peritoneal metastasis from colorectal cancer: a review-an English version. J Anus Rectum Colon (2022) 6(4):197–202. doi: 10.23922/jarc.2022-045 36348949 PMC9613417

[10] Kanda M Kodera Y. Molecular mechanisms of peritoneal dissemination in gastric cancer. World J Gastroenterol (2016) 22(30):6829–40. doi: 10.3748/wjg.v22.i30.6829 PMC4974582 27570420

[11] Arneth B. Tumor microenvironment. Medicina (Kaunas) (2019) 56(1):15. doi: 10.3390/me-dicina56010015 31906017 PMC7023392

[12] Korbecki J Kojder K Kapczuk P Kupnicka P Gawrońska-Szklarz B Gutowska I. The effect of hypoxia on the expression of CXC chemokines and CXC chemokine receptors-a review of literature. Int J Mol Sci (2021) 22(2):843. doi: 10.3390/ijms22020843 33467722 PMC7830156

[13] Hanahan D Weinberg RA. Hallmarks of cancer: the next generation. Cell (2011) 144(5):646–74. doi: 10.1016/j.cell.2011.02.013 21376230

[14] Rasool M Natesan Pushparaj P Karim S. Overexpression of *CXCL8* gene in Saudi colon cancer patients. Saudi J Biol Sci (2021) 28(11):6045–9. doi: 10.1016/j.sjbs.2021.09.031 PMC8568844 34764737

[15] Liu Z Wu X Tian Y Zhang W Qiao S Xu W. H. pylori infection induces CXCL8 expression and promotes gastric cancer progress through downregulating KLF4. Mol Carcinog (2021) 60(8):524–37. doi: 10.1002/mc.23309 34038586

[16] Ruffini PA. The CXCL8-CXCR1/2 axis as a therapeutic target in breast cancer stem-like cells. Front Oncol (2019) 9:40. doi: 10.3389/fonc.2019.00040 30788286 PMC6373429

[17] Adekoya TO Richardson RM. Cytokines and chemokines as mediators of prostate cancer metastasis. Int J Mol Sci (2020) 21(12):4449. doi: 10.3390/ijms21124449 32585812 PMC7352203

[18] Samanta D Gilkes DM Chaturvedi P Xiang L Semenza GL. Hypoxia-inducible factors are required for chemotherapy resistance of breast cancer stem cells. Proc Natl Acad Sci USA (2014) 111(50):E5429–38. doi: 10.1073/pnas.1421438111 PMC4273385 25453096

[19] Pawluczuk E Łukaszewicz-Zając M Gryko M Kulczyńska-Przybik A Mroczko B. Serum CXCL8 and its specific receptor (CXCR2) in gastric cancer. Cancers (Basel) (2021) 13(20):5186. doi: 10.3390/cancers13205186 34680333 PMC8534112

[20] Baggiolini M Walz A Kunkel SL. Neutrophil-activating peptide-1/interleukin 8, a- novel cytokine that activates neutrophils. J Clin Invest (1989) 84(4):1045–9. doi: 10.1172/JCI114265 PMC329758 2677047

[21] Le Y Zhou Y Iribarren P Wang J. Chemokines and chemokine receptors: their manifold roles in homeostasis and disease. Cell Mol Immunol (2004) 1(2):95–104. 16212895

[22] Aoki M Fujishita T. Oncogenic roles of the PI3K/AKT/mTOR axis. Curr Top Microbiol Immunol (2017) 407:153–89. doi: 10.1007/82_2017_6 28550454

[23] Bertagnolo V Benedusi M Brugnoli F Lanuti P Marchisio M Querzoli P. Phospholipase c-beta 2 promotes mitosis and migration of human breast cancer-derived cells. Carcinogenesis (2007) 28(8):1638–45. doi: 10.1093/carcin/bgm078 17429106

[24] Pencik J Pham HT Schmoellerl J Javaheri T Schlederer M Culig Z. JAK-STAT signaling in cancer: from cytokines to non-coding genome. Cytokine (2016) 87:26–36. doi: 10.1016/j.cyto.2016.06.017 27349799 PMC6059362

[25] Dohlman HG Campbell SL. Regulation of large and small G proteins by ubiquitination. J Biol Chem (2019) 294(49):18613–23. doi: 10.1074/jbc.REV119.011068 PMC6901297 31645437

[26] Baggiolini M. CXCL8 - the first chemokine. Front Immunol (2015) 6:285. doi: 10.3389/fimmu.2015.00285 26106391 PMC4459227

[27] Liu Q Li A Yu S Qin S Han N Pestell RG. DACH1 antagonizes CXCL8 to repress tumorigenesis of lung adenocarcinoma and improve prognosis. J Hematol Oncol (2018) 11(1):53. doi: 10.1186/s13045-018-0597-1 29636079 PMC5894143

[28] Mukaida N Shiroo M Matsushima K. Genomic structure of the human monocyte-derived neutrophil chemotactic factor IL-8. J Immunol (1989) 143(4):1366–71. doi: 10.4049/jimmunol.143.4.1366 2663993

[29] Hébert CA Baker JB. Interleukin-8: a review. Cancer Invest (1993) 11(6):743–50. doi: 10.3109/07357909309046949 8221207

[30] Peveri P Walz A Dewald B Baggiolini M. A novel neutrophil-activating factor produced by human mononuclear phagocytes. J Exp Med (1988) 167(5):1547–59. doi: 10.1084/jem.167.5.1547 PMC2188939 2835419

[31] Strieter RM Kunkel SL Showell HJ Marks RM. Monokine-induced gene expression of a human endothelial cell-derived neutrophil chemotactic factor. Biochem Biophys Res Commun (1988) 156(3):1340–5. doi: 10.1016/s0006-291x(88)80779-4 3263857

[32] Kwon OJ Au BT Collins PD Adcock IM Mak JC Robbins RR. Tumor necrosis factor-induced interleukin-8 expression in cultured human airway epithelial cells. Am J Physiol (1994) 267(4 Pt 1):L398–405. doi: 10.1152/ajplung.1994.267.4.L398 7943343

[33] Wanninger J Neumeier M Weigert J Bauer S Weiss TS Schäffler A. Adiponectin-stimulated CXCL8 release in primary human hepatocytes is regulated by ERK1/ERK2, p38 MAPK, NF-kappaB, and STAT3 signaling pathways. Am J Physiol Gastrointest Liver Physiol (2009) 297(3):G611–8. doi: 10.1152/ajpgi.90644.2008 19608729

[34] Ning Y Manegold PC Hong YK Zhang W Pohl A Lurje G. Interleukin-8 is associated with proliferation, migration, angiogenesis and chemosensitivity *in vitro* and *in vivo* in colon cancer cell line models. Int J Cancer (2011) 128(9):2038–49. doi: 10.1002/ijc.25562 PMC3039715 20648559

[35] Mishra P Banerjee D Ben-Baruch A. Chemokines at the crossroads of tumor-fibr-oblast interactions that promote malignancy. J Leukoc Biol (2011) 89(1):31–9. doi: 10.1189/jlb.0310182 20628066

[36] Dmitrzak-Weglarz M Szczepankiewicz A Rybakowski J Kapelski P Bilska K Skibinska M. Transcriptomic profiling as biological markers of depression - a pilot study in unipolar and bipolar women. World J Biol Psychiatry (2021) 22(10):744–56. doi: 10.1080/15622975.2021.1907715 33821765

[37] Bertelsen T Iversen L Johansen C. The human IL-17A/F heterodimer regulates psoriasis-associated genes through IκBζ. Exp Dermatol (2018) 27(9):1048–52. doi: 10.1111/exd.13722 29938836

[38] Clifford RL Patel JK John AE Tatler AL Mazengarb L Brightling CE. CXCL8 histone H3 acetylation is dysfunctional in airway smooth muscle in asthma: regulation by BET. Am J Physiol Lung Cell Mol Physiol (2015) 308(9):L962–72. doi: 10.1152/ajplung.00021.2015 PMC4421784 25713319

[39] Gambari R Borgatti M Bezzerri V Nicolis E Lampronti I Dechecchi MC. Decoy oligodeoxyribonucleotides and peptide nucleic acids-DNA chimeras targeting nuclear factor kappa-b: inhibition of IL-8 gene expression in cystic fibrosis cells infected with pseudomonas aeruginosa. Biochem Pharmacol (2010) 80(12):1887–94. doi: 10.1016/j.bcp.2010.06.047 20615393

[40] Ning Y Lenz HJ. Targeting IL-8 in colorectal cancer. Expert Opin Ther Targets (2012) 16(5):491–7. doi: 10.1517/14728222.2012.677440 22494524

[41] Kosmopoulos M Christofides A Drekolias D Zavras PD Gargalionis AN Piperi C. Critical role of IL-8 targeti-ng in gliomas. Curr Med Chem (2018) 25(17):1954–67. doi: 10.2174/0929867325666171129125712 29189120

[42] Guo Y Zang Y Lv L Cai F Qian T Zhang G. IL-8 promotes proliferation and inhibition of apoptosis via STAT3/AKT/NF-κB pathway in prostate cancer. Mol Med Rep (2017) 16(6):9035–42. doi: 10.3892/mmr.2017.7747 29039490

[43] Yang A Lu Y Xing J Li Z Yin X Dou C. IL-8 enhances therapeutic effects of BMSCs on bone regeneration via CXCR2-mediated PI3k/Akt signaling pathway. Cell Physiol Biochem (2018) 48(1):361–70. doi: 10.1159/000491742 30016780

[44] Ha H Debnath B Neamati N. Role of the CXCL8-CXCR1/2 axis in cancer and inflammatory diseases. Theranostics (2017) 7(6):1543–88. doi: 10.7150/thno.15625 PMC5436513 28529637

[45] Bi H Zhang Y Wang S Fang W He W Yin L. Interleukin-8 promotes cell migration via CXCR1 and CXCR2 in liver cancer. Oncol Lett (2019) 18(4):4176–84. doi: 10.3892/ol.2019.10735 PMC6732969 31516616

[46] Murphy PM Tiffany HL. Cloning of complementary DNA encoding a functional human interleukin-8 receptor. Science (1991) 253(5025):1280–3. doi: 10.1126/science.1891716 1891716

[47] Holmes WE Lee J Kuang WJ Rice GC Wood WI. Structure and functional expression of a human interleukin-8 receptor. Science (1991) 253(5025):1278–80. doi: 10.1126/science.1840701 1840701

[48] Park SH Das BB Casagrande F Tian Y Nothnagel HJ Chu M. Structure of the chemokine receptor CXCR1 in phospholipid bilayers. Nature (2012) 491(7426):779–83. doi: 10.1038/nature11580 PMC3700570 23086146

[49] Yung MM Tang HW Cai PC Leung TH Ngu SF Chan KK. GRO-α and IL-8 enhance ovarian cancer metastatic n potential via the CXCR2-mediated TAK1/NFκB signaling cascade. Theranostics (2018) 8(5):1270–85. doi: 10.7150/thno.22536 PMC5835935 29507619

[50] Liu Q Li A Tian Y Wu JD Liu Y Li T. The CXCL8-CXCR1/2 pathways in cancer. Cytokine Growth Factor Rev (2016) 31:61–71. doi: 10.1016/j.cytogfr.2016.08.002 27578214 PMC6142815

[51] Tavares LP Garcia CC Machado MG Queiroz-Junior CM Barthelemy A Trottein F. CXCR1/2 antagonism is protective during influenza and post-influenza pneumococcal infection. Front Immunol (2017) 8:1799. doi: 10.3389/fimmu.2017.01799 29326698 PMC5733534

[52] Chuntharapai A Lee J Hébert CA Kim KJ. Monoclonal antibodies detect different distribution patterns of IL-8 receptor a and IL-8 receptor b on human peripheral blood leukocytes. J Immunol (1994) 153(12):5682–8. doi: 10.4049/jimmunol.153.12.5682 7527448

[53] Zuccari DA Leonel C Castro R Gelaleti GB Jardim BV Moscheta MG. An immunohistochemical study of interleukin-8 (IL-8) in breast cancer. Acta Histochem (2012) 114(6):571–6. doi: 10.1016/j.acthis.2011.10.007 22244449

[54] Singh S Sadanandam A Varney ML Nannuru KC Singh RK. Small interfering RNA-mediated CXCR1 or CXCR2 knock-down inhibits melanoma tumor growth and invasion. Int J Cancer (2010) 126(2):328–36. doi: 10.1002/ijc.24714 PMC2794956 19585580

[55] Li A Varney ML Singh RK. Expression of interleukin 8 and its receptors in human colon carcinoma cells with different metastatic potentials. Clin Cancer Res (2001) 7(10):3298–304. 11595728

[56] Franz JM Portela P Salim PH Berger M Fernando Jobim L Roesler R. CXCR2 + 1208 CT genotype may predict earlier clinical stage at diagnosis in patients with prostate cancer. Cytokine (2017) 97:193–200. doi: 10.1016/j.cyto.2017.06.001 28668699

[57] Li Z Wang Y Dong S Ge C Xiao Y Li R. Association of CXCR1 and 2 expressions with gastric cancer metastasis in *ex vivo* and tumor cell invasion *in vitro* . Cytokine (2014) 69(1):6–13. doi: 10.1016/j.cyto.2014.05.004 25022956

[58] Damaj BB McColl SR Mahana W Crouch MF Naccache PH. Physical association of Gi2alpha with interleukin-8 receptors. J Biol Chem (1996) 271(22):12783–9. doi: 10.1074/jbc.271.22.12783 8662698

[59] Chen QY Costa M. PI3K/Akt/mTOR signaling pathway and the biphasic effect of arsenic in carcinogenesis. Mol Pharmacol (2018) 94(1):784–92. doi: 10.1124/mol.118.112268 PMC5994485 29769245

[60] Tan AC. Targeting the PI3K/Akt/mTOR pathway in non-small cell lung cancer (NSCLC). Thorac Cancer (2020) 11(3):511–8. doi: 10.1111/1759-7714.13328 PMC7049515 31989769

[61] Fresno Vara JA Casado E de Castro J Cejas P Belda-Iniesta C González-Barón M. PI3K/Akt signalling pathway and cancer. Cancer Treat Rev (2004) 30(2):193–204. doi: 10.1016/j.ctrv.2003.07.007 15023437

[62] Jiang BH Liu LZ. PI3K/PTEN signaling in angiogenesis and tumorigenesis. Adv Cancer Res (2009) 102:19–65. doi: 10.1016/S0065-230X(09)02002-8 19595306 PMC2933405

[63] Pompura SL Dominguez-Villar M. The PI3K/AKT signaling pathway in regulatory T-cell development, stability, and function. J Leukoc Biol (2018) 103(6):1065–76. doi: 10.1002/JLB.2MIR0817-349R 29357116

[64] Ringe J Strassburg S Neumann K Endres M Notter M Burmester GR. Towards *in situ* tissue repair: human mesenchymal stem cells express chemokine receptors CXCR1, CXCR2 and CCR2, and migrate upon stimulation with CXCL8 but not CCL2. J Cell Biochem (2007) 101(1):135–46. doi: 10.1002/jcb.21172 17295203

[65] Choi JW Shin S Lee CY Lee J Seo HH Lim S. Rapid induction of osteogenic markers in mesenchymal stem cells by adipose-derived stromal vascular fraction cells. Cell Physiol Biochem (2017) 44(1):53–65. doi: 10.1159/000484582 29131029

[66] Hu L Hofmann J Jaffe RB. Phosphatidylinositol 3-kinase mediates angiogenesis and vascular permeability associated with ovarian carcinoma. Clin Cancer Res (2005) 11(22):8208–12. doi: 10.1158/1078-0432.CCR-05-0206 16299254

[67] Karar J Maity A. PI3K/AKT/mTOR pathway in angiogenesis. Front Mol Neurosci (2011) 4:51. doi: 10.3389/fnmol.2011.00051 22144946 PMC3228996

[68] Jiang H Wang X Miao W Wang B Qiu Y. CXCL8 promotes the invasion of human osteosarcoma cells by regulation of PI3K/Akt signaling pathway. APMIS (2017) 125(9):773–80. doi: 10.1111/apm.12721 28736978

[69] Shen T Yang Z Cheng X Xiao Y Yu K Cai X. CXCL8 induces epithelial-mesenchymal transition in colon cancer cells via the PI3K/Akt/NF-κB signaling pathway. Oncol Rep (2017) 37(4):2095–100. doi: 10.3892/or.2017.5453 28259918

[70] Danielsen SA Cekaite L Ågesen TH Sveen A Nesbakken A Thiis-Evensen E. Phospholipase c isozymes are deregulated in colorectal cancer–insights gained from gene set enrichment analysis of the transcriptome. PloS One (2011) 6(9):e24419. doi: 10.1371/journal.pone.0024419 21909432 PMC3164721

[71] Nishizuka Y. Protein kinase c and lipid signaling for sustained cellular responses. FASEB J (1995) 9(7):484–96. doi: 10.1096/fasebj.9.7.7737456 7737456

[72] Kelley GG Reks SE Ondrako JM Smrcka AV. Phospholipase c(epsilon): a novel ras effector. EMBO J (2001) 20(4):743–54. doi: 10.1093/emboj/20.4.743 PMC145421 11179219

[73] Newton AC. Protein kinase c: perfectly balanced. Crit Rev Biochem Mol Biol (2018) 53(2):208–30. doi: 10.1080/10409238.2018.1442408 PMC5901981 29513138

[74] Nishizuka Y. Intracellular signaling by hydrolysis of phospholipids and activation of protein kinase c. Science (1992) 258(5082):607–14. doi: 10.1126/science.1411571 1411571

[75] Musashi M Ota S Shiroshita N. The role of protein kinase c isoforms in cell proliferation and apoptosis. Int J Hematol (2000) 72(1):12–9. 10979203

[76] Kanemura S Tsuchiya A Kanno T Nakano T Nishizaki T. Phosphatidylinositol induces caspase-independent apoptosis of malignant pleural mesothelioma cells by accumulating AIF in the nucleus. Cell Physiol Biochem (2015) 36(3):1037–48. doi: 10.1159/000430277 26112407

[77] Di Paolo G De Camilli P. Phosphoinositides in cell regulation and membrane dynamics. Nature (2006) 443(7112):651–7. doi: 10.1038/nature05185 17035995

[78] Bill CA Vines CM. Phospholipase c. Adv Exp Med Biol (2020) 1131:215–42. doi: 10.1007/978-3-030-12457-1_9 PMC7790445 31646512

[79] Böhm D Schwegler H Kotthaus L Nayernia K Rickmann M Köhler M. Disruption of PLC-beta 1-mediated signal transduction in mutant mice causes age-dependent hippocampal mossy fiber sprouting and neurodegeneration. Mol Cell Neurosci (2002) 21(4):584–601. doi: 10.1006/mcne.2002.1199 12504592

[80] Mende U Kagen A Meister M Neer EJ. Signal transduction in atria and ventricles of mice with transient cardiac expression of activated G protein alpha(q). Circ Res (1999) 85(11):1085–91. doi: 10.1161/01.res.85.11.1085 10571540

[81] Martelli AM Evangelisti C Nyakern M Manzoli FA. Nuclear protein kinase c. Biochim Biophys Acta (2006) 1761(5-6):542–51. doi: 10.1016/j.bbalip.2006.02.009 16574477

[82] Chen J Wang W Zhang T Ji J Qian Q Lu L. Differential expression of phospholipase c epsilon 1 is associated with chronic atrophic gastritis and gastric cancer. PloS One (2012) 7(10):e47563. doi: 10.1371/journal.pone.0047563 23077637 PMC3471869

[83] Owusu Obeng E Rusciano I Marvi MV Fazio A Ratti S Follo MY. Phosphoinositide-dependent signaling in cancer: a focus on phospholipase c isozymes. Int J Mol Sc (2020) 21(7):2581. doi: 10.3390/ijms21072581 32276377 PMC7177890

[84] Koss H Bunney TD Behjati S Katan M. Dysfunction of phospholipase cγ in immune disorders and cancer. Trends Biochem Sci (2014) 39(12):603–11. doi: 10.1016/j.tibs.2014.09.004 25456276

[85] Fu XT Dai Z Song K Zhang ZJ Zhou ZJ Zhou SL. Macrophage-secreted IL-8 induces epithelial-mesenchymaltransition in hepatocellular carcinoma cells by activating the JAK2/STAT3/Snail pathway. Int J Oncol (2015) 46(2):587–96. doi: 10.3892/ijo.2014.2761 25405790

[86] Owen KL Brockwell NK Parker BS. JAK-STAT signaling: a double-edged sword of immune regulation and cancer progression. Cancers (Basel) (2019) 11(12):2002. doi: 10.3390/cancers11122002 31842362 PMC6966445

[87] Banerjee S Biehl A Gadina M Hasni S Schwartz DM. JAK-STAT signaling as a target for inflammatory and autoimmune diseases: current and future prospects. Drugs (2017) 77(5):521–46. doi: 10.1007/s40265-017-0701-9 PMC7102286 28255960

[88] Michailova K Wassilev W Wedel T. Scanning and transmission electron microscopic study of visceral and parietal peritoneal regions in the rat. Ann Anat (1999) 181(3):253–60. doi: 10.1016/S0940-9602(99)80040-5 10363107

[89] Andrews PM Porter KR. The ultrastructural morphology and possible functional significance of mesothelial microvilli. Anat Rec (1973) 177(3):409–26. doi: 10.1002/ar.1091770307 4127780

[90] Sugarbaker PH. Peritoneum as the first-line of defense in carcinomatosis. J Surg Oncol (2007) 95(2):93–6. doi: 10.1002/jso.20676 17262739

[91] Lemoine L Sugarbaker P van der Speeten K. Pathophysiology of colorectal peritoneal carcinomatosis: role of the peritoneum. World J Gastroenterol (2016) 22(34):7692–707. doi: 10.3748/wjg.v22.i34.7692 PMC5016368 27678351

[92] Mutsaers SE Wilkosz S. Structure and function of mesothelial cells. Cancer Treat Res (2007) 134:1–19. doi: 10.1007/978-0-387-48993-3_1 17633044

[93] van der Wal JB Jeekel J. Biology of the peritoneum in normal homeostasis and after surgical trauma. Colorectal Dis (2007) 9 Suppl 2:9–13. doi: 10.1111/j.1463-1318.2007.01345.x 17824965

[94] Mikuła-Pietrasik J Uruski P Tykarski A Książek K. The peritoneal “soil” for a cancerous “seed”: a comprehensive review of the pathogenesis of intraperitoneal cancer metastases. Cell Mol Life Sci (2018) 75(3):509–25. doi: 10.1007/s00018-017-2663-1 PMC5765197 28956065

[95] Blackburn SC Stanton MP. Anatomy and physiology of the peritoneum. Semin Pediatr Surg (2014) 23(6):326–30. doi: 10.1053/j.sempedsurg.2014.06.002 25459436

[96] Aguirre AR Abensur H. Fisiologia do transporte de fluidos e solutos atraves da membrana peritoneal [Physiology of fluid and solute transport across the peritoneal membrane]. J Bras Nefrol (2014) 36(1):74–9. doi: 10.5935/0101-2800.2014001 24676618

[97] Warn R Harvey P Warn A Foley-Comer A Heldin P Versnel M. HGF/SF induces mesothelial cell migration and proliferation by autocrine and paracrine pathways. Exp Cell Res (2001) 267(2):258–66. doi: 10.1006/excr.2001.5240 11426944

[98] Mutsaers SE. Mesothelial cells: their structure, function and role in serosal repair. Respirology (2002) 7(3):171–91. doi: 10.1046/j.1440-1843.2002.00404.x 12153683

[99] Riese J Denzel C Zowe M Mehler C Hohenberger W Haupt W. Secretion of IL-6, monocyte chemoattractant protein-1, macrophage inflammatory protein-1alpha, and TNFalpha by cultured intact human peritoneum. Eur Surg Res (1999) 31(3):281–8. doi: 10.1159/000008704 10352357

[100] Kawanishi K. Diverse properties of the mesothelial cells in health and disease. Pleura Peritoneum (2016) 1(2):79–89. doi: 10.1515/pp-2016-0009 30911611 PMC6386382

[101] Yao V Platell C Hall JC. Role of peritoneal mesothelial cells in peritonitis. Br J Surg (2003) 90(10):1187–94. doi: 10.1002/bjs.4373 14515285

[102] Aufricht C Neuhofer W Topley N Wörnle M. Peritoneal infection and inflammation. Mediators Inflamm (2012) 2012:456985. doi: 10.1155/2012/456985 22719177 PMC3376786

[103] Martin P Hopkinson-Woolley J McCluskey J. Growth factors and cutaneous wound repair. Prog Growth Factor Res (1992) 4(1):25–44. doi: 10.1016/0955-2235(92)90003-z 1325207

[104] Visser CE Brouwer-Steenbergen JJ Schadee-Eestermans IL Meijer S Krediet RT Beelen RH. Ingestion of staphylococcus aureus, staphylococcus epidermidis, and escherichia coli by human peritoneal mesothelial cells. Infect Immun (1996) 64(8):3425–8. doi: 10.1128/iai.64.8.3425-3428.1996 PMC174242 8757887

[105] Visser CE Steenbergen JJ Betjes MG Meijer S Arisz L Hoefsmit EC. Interleukin-8 production by human mesothelial cells after direct stimulation with staphylococci. Infect Immun (1995) 63(10):4206–9. doi: 10.1128/iai.63.10.4206-4209.1995 PMC173597 7558346

[106] Witowski J Pawlaczyk K Breborowicz A Scheuren A Kuzlan-Pawlaczyk M Wisniewska J. IL-17 stimulates intraperitoneal neutrophil infiltration through the release of GRO alpha chemokine from mesothelial cells. J Immunol (2000) 165(10):5814–21. doi: 10.4049/jimmunol.165.10.5814 11067941

[107] Fujino S Yokoyama A Kohno N Hiwada K. Interleukin 6 is an autocrine growth factor for normal human pleural mesothelial cells. Am J Respir Cell Mol Biol (1996) 14(6):508–15. doi: 10.1165/ajrcmb.14.6.8652179 8652179

[108] Wijffels JF Hendrickx RJ Steenbergen JJ Eestermans IL Beelen RH. Milky spots in the mouse omentum may play an important role in the origin of peritoneal macrophages. Res Immunol (1992) 143(4):401–9. doi: 10.1016/s0923-2494(05)80072-0 1518954

[109] Fialová A Partlová S Sojka L Hromádková H Brtnický T Fučíková J. Dynamics of T-cell infiltration during the course of ovarian cancer: the gradual shift from a Th17 effector cell response to a predominant infiltration by regulatory T-cells. Int J Cancer (2013) 132(5):1070–9. doi: 10.1002/ijc.27759 22865582

[110] Wertel I Surówka J Polak G Barczyński B Bednarek W Jakubowicz-Gil J. Macrophage-derived chemokine CCL22 and regulatory T cells in ovarian cancer patients. Tumour Biol (2015) 36(6):4811–7. doi: 10.1007/s13277-015-3133-8 PMC4529457 25647263

[111] Gatla HR Zou Y Uddin MM Vancurova I. Epigenetic regulation of interleukin-8 expression by class I HDAC and CBP in ovarian cancer cells. Oncotarget (2017) 8(41):70798–810. doi: 10.18632/oncotarget.19990 PMC5642595 29050320

[112] Runyon BA Hoefs JC Morgan TR. Ascitic fluid analysis in malignancy-related ascites. Hepatology (1988) 8(5):1104–9. doi: 10.1002/hep.1840080521 3417231

[113] Berek JS Renz M Kehoe S Kumar L Friedlander M. Cancer of the ovary, fallopian tube, and peritoneum: 2021 update. Int J Gynaecol Obstet (2021) 155 Suppl 1(Suppl 1):61–85. doi: 10.1002/ijgo.13878 34669199 PMC9298325

[114] Lengyel E. Ovarian cancer development and metastasis. Am J Pathol (2010) 177(3):1053–64. doi: 10.2353/ajpath.2010.100105 PMC2928939 20651229

[115] Sodek KL Murphy KJ Brown TJ Ringuette MJ. Cell-cell and cell-matrix dynamics in intraperitoneal cancer metastasis. Cancer Metastasis Rev (2012) 31(1-2):397–414. doi: 10.1007/s10555-012-9351-2 22527451 PMC3350631

[116] Klymenko Y Nephew KP. Epigenetic crosstalk between the tumor microenvironment and ovarian cancer cells: a therapeutic road less traveled. Cancers (Basel) (2018) 10(9):295. doi: 10.3390/cancers10090295 30200265 PMC6162502

[117] Benamro F Sartorius B Clarke DL Anderson F Loots E Olinger L. The spectrum of gastric cancer as seen in a large quaternary hospital in KwaZulu-natal, south Africa. S Afr Med J (2017) 107(2):130–3. doi: 10.7196/SAMJ.2017.v107i2.11383 28220739

[118] Saito H Fushida S Harada S Miyashita T Oyama K Yamaguchi T. Importance of human peritoneal mesothelial cells in the progression, fibrosis, and control of gastric cancer: inhibition of growth and fibrosis by tranilast. Gastric Cancer (2018) 21(1):55–67. doi: 10.1007/s10120-017-0726-5 28540637 PMC5741788

[119] Halkia E Spiliotis J Sugarbaker P. Diagnosis and management of peritoneal metastases from ovarian cancer. Gastroenterol Res Pract (2012) 2012:541842. doi: 10.1155/2012/541842 22888339 PMC3408715

[120] Paget S. The distribution of secondary growths in cancer of the breast. 1889. Cancer Metastasis Rev (1889) 8(2):98–101. doi: 10.1016/S0140-6736(00)49915-0 2673568

[121] Jayne D. Molecular biology of peritoneal carcinomatosis. Cancer Treat Res (2007) 134:21–33. doi: 10.1007/978-0-387-48993-3_2 17633045

[122] Pascual-Antón L Cardeñes B Sainz de la Cuesta R González-Cortijo L López-Cabrera M Cabañas C. Mesothelial-to-Mesenchymal transition and exosomes in peritoneal metastasis of ovarian cancer. Int J Mol Sci (2021) 22(21):11496. doi: 10.3390/ijms222111496 34768926 PMC8584135

[123] Wang Z Chen JQ Liu JL Tian L. Issues on peritoneal metastasis of gastric cancer: an update. World J Surg Oncol (2019) 17(1):215. doi: 10.1186/s12957-019-1761-y 31829265 PMC6907197

[124] Yilmaz M Christofori G. EMT, the cytoskeleton, and cancer cell invasion. Cancer Metastasis Rev (2009) 28(1-2):15–33. doi: 10.1007/s10555-008-9169-0 19169796

[125] Hirohashi S. Inactivation of the e-cadherin-mediated cell adhesion system in human cancers. Am J Pathol (1998) 153(2):333–9. doi: 10.1016/S0002-9440(10)65575-7 PMC1852964 9708792

[126] Principe S Jones EE Kim Y Sinha A Nyalwidhe JO Brooks J. In-depth proteomic analyses of exosomes isolated from expressed prostatic secretions in urine. Proteomics (2013) 13(10-11):1667–71. doi: 10.1002/pmic.201200561 PMC3773505 23533145

[127] Chiasserini D van Weering JR Piersma SR Pham TV Malekzadeh A Teunissen CE. Proteomic analysis of cerebrospinal fluid extracellular vesicles: a comprehensive dataset. J Proteomics (2014) 106:191–204. doi: 10.1016/j.jprot.2014.04.028 24769233

[128] Lee JC Kang SU Jeon Y Park JW You JS Ha SW. Protein l-isoaspartyl methyltransferase regulates p53 activity. Nat Commun (2012) 3:927. doi: 10.1038/ncomms1933 22735455 PMC3621463

[129] Zhang J Li Y Liu H Zhang J Wang J Xia J. Genome-wide CRISPR/Cas9 library screen identifies PCMT1 as a critical driver of ovarian cancer metastasis. J. Exp Clin Cancer Res (2022) 41(1):24. doi: 10.1186/s13046-022-02242-3 PMC8760697 35033172

[130] Mikuła-Pietrasik J Uruski P Sosińska P Maksin K Piotrowska-Kempisty H Kucińska M. Senescent peritoneal mesothelium creates a niche for ovarian cancer metastases. Cell Death Dis (2016) 7(12):e2565. doi: 10.1038/cddis.2016.417 28032864 PMC5261005

[131] Ksiazek K Mikula-Pietrasik J Korybalska K Dworacki G Jörres A Witowski J. Senescent peritoneal mesothelial cells promote ovarian cancer cell adhesion: the role of oxidative stress-induced fibronectin. Am J Pathol (2009) 174(4):1230–40. doi: 10.2353/ajpath.2009.080613 PMC2671356 19246646

[132] Mikuła-Pietrasik J Sosińska P Maksin K Kucińska MG Piotrowska H Murias M. Colorectal cancer-promoting activity of the senescent peritoneal mesothelium. Oncotarget (2015) 6(30):29178–95. doi: 10.18632/oncotarget.4932 PMC4745719 26284488

[133] Ksiazek K Mikuła-Pietrasik J Catar R Dworacki G Winckiewicz M Frydrychowicz M. Oxidative stress-dependent increase in ICAM-1 expression promotes adhesion of colorectal and pancreatic cancers to the senescent peritoneal mesothelium. Int J Cancer (2010) 127(2):293–303. doi: 10.1002/ijc.25036 19904754

[134] Bracke ME. Role of adhesion molecules in locoregional cancer spread. Cancer Treat Res (2007) 134:35–49. doi: 10.1007/978-0-387-48993-3_3 17633046

[135] Strobel T Cannistra SA. Beta1-integrins partly mediate binding of ovarian cancer cells to peritoneal mesothelium *in vitro* . Gynecol Oncol (1999) 73(3):362–7. doi: 10.1006/gyno.1999.5388 10366461

[136] Nakashio T Narita T Akiyama S Kasai Y Kondo K Ito K. Adhesion molecules and TGF-beta1 are involved in the peritoneal dissemination of NUGC-4 human gastric cancer cells. Int J Cancer (1997) 70(5):612–8. doi: 10.1002/(SICI)1097-0215(19970304)70:5<612::AID-IJC20>3.0.CO;2-D 9052764

[137] Kawamura T Endo Y Yonemura Y Nojima N Fujita H Fujimura T. Significance of integrin alpha2/beta1 in peritoneal dissemination of a human gastric cancer xenograft model. Int J Oncol (2001) 18(4):809–15. 11251178

[138] Wagner BJ Löb S Lindau D Hörzer H Gückel B Klein G. Simvastatin reduces tumor cell adhesion to human peritoneal mesothelial cells by decreased expression of VCAM-1 and β1 integrin. Int J Oncol (2011) 39(6):1593–600. doi: 10.3892/ijo.2011.1167 21874229

[139] Aruffo A Stamenkovic I Melnick M Underhill CB Seed B. CD44 is the principal cell surface receptor for hyaluronate. Cell (1990) 61(7):1303–13. doi: 10.1016/0092-8674(90)90694-a 1694723

[140] Rump A Morikawa Y Tanaka M Minami S Umesaki N Takeuchi M. Binding of ovarian cancer antigen CA125/MUC16 to mesothelin mediates cell adhesion. J Biol Chem (2004) 279(10):9190–8. doi: 10.1074/jbc.M312372200 14676194

[141] Sako A Kitayama J Yamaguchi H Kaisaki S Suzuki H Fukatsu K. Vascular endothelial growth factor synthesis by human omental mesothelial cells is augmented by fibroblast growth factor-2: possible role of mesothelial cell on the development of peritoneal metastasis. J Surg Res (2003) 115(1):113–20. doi: 10.1016/s0022-4804(03)00307-x 14572781

[142] Lv ZD Wang HB Dong Q Kong B Li JG Yang ZC. Mesothelial cells differentiate into fibroblast-like cells under the scirrhous gastric cancer microenvironment and promote peritoneal carcinomatosis *in vitro* and *in vivo* . Mol Cell Biochem (2013) 377(1-2):177–85. doi: 10.1007/s11010-013-1583-0 23392771

[143] Bhowmick NA Neilson EG Moses HL. Stromal fibroblasts in cancer initiation and progression. Nature (2004) 432(7015):332–7. doi: 10.1038/nature03096 PMC3050735 15549095

[144] Gianni-Barrera R Trani M Reginato S Banfi A. To sprout or to split? VEGF, notch and vascular morphogenesis. Biochem Soc Trans (2011) 39(6):1644–8. doi: 10.1042/BST20110650 22103501

[145] Yamaguchi T Fushida S Yamamoto Y Tsukada T Kinoshita J Oyama K. Tumor-associated macrophages of the M2 phenotype contribute to progression in gastric cancer with peritoneal dissemination. Gastric Cancer (2016) 19(4):1052–65. doi: 10.1007/s10120-015-0579-8 PMC5034006 26621525

[146] Alfaro C Sanmamed MF Rodríguez-Ruiz ME Teijeira Á Oñate C González Á. Interleukin-8 in cancer pathogenesis, treatment and follow-up. Cancer Treat Rev (2017) 60:24–31. doi: 10.1016/j.ctrv.2017.08.004 28866366

[147] Jia X Lu M Rui C. Consensus-expressed CXCL8 and MMP9 identified by meta-analyzed perineural invasion gene signature in gastric cancer microarray data. Front Genet (2019) 10:851. doi: 10.3389/fgene.2019.00851 31681401 PMC6798046

[148] Smycz-Kubańska M Stępień S Gola JM Kruszniewska-Rajs C Wendlocha D Królewska-Daszczyńska P. Analysis of CXCL8 and its receptors CXCR1/CXCR2 at the mRNA level in neoplastic tissue, as well as in serum and peritoneal fluid in patients with ovarian cance. Mol Med Rep (2022) 26(4):296. doi: 10.3892/mmr.2022.12812 35920183 PMC9435018

[149] Xiong X Liao X Qiu S Xu H Zhang S Wang S. CXCL8 in tumor biology and its implications for clinical translation. Front Mol Biosci (2022) 9:723846. doi: 10.3389/fmolb.2022.723846 35372515 PMC8965068

[150] Pasquier J Vidal F Hoarau-Véchot J Bonneau C Daraï E Touboul C. Surgical peritoneal stress creates a pro-metastatic niche promoting resistance to apoptosis via IL-8. J Transl Med (2018) 16(1):271. doi: 10.1186/s12967-018-1643-z 30285881 PMC6171219

[151] Raman D Sai J Hawkins O Richmond A. Adaptor protein2 (AP2) orchestrates CXCR2-mediated cell migration. Traffic (2014) 15(4):451–69. doi: 10.1111/tra.12154 PMC3966550 24450359

[152] Asfaha S Dubeykovskiy AN Tomita H Yang X Stokes S Shibata W. Mice that express human interleukin-8 have increased mobilization of immature myeloid cells, which exacerbates inflammation and accelerates colon carcinogenesis. Gastroenterology (2013) 144(1):155–66. doi: 10.1053/j.gastro.2012.09.057 PMC3990262 23041326

[153] Dolgin E. BMS bets on targeting IL-8 to enhance cancer immunotherapies. Nat Biotechnol (2016) 34(10):1006–7. doi: 10.1038/nbt1016-1006 27727222

[154] David JM Dominguez C Hamilton DH Palena C. The IL-8/IL-8R axis: a double agent in tumor immune resistance. Vaccines (Basel) (2016) 4(3):22. doi: 10.3390/vaccines4030022 27348007 PMC5041016

[155] Li XH Qu JQ Yi H Zhang PF Yi HM Wan XX. Integrated analysis of differential miRNA and mRNA expression profiles in human radioresistant and radiosensitive nasopharyngeal carcinoma cells. PloS One (2014) 9(1):e87767. doi: 10.1371/journal.pone.0087767 24498188 PMC3909230

[156] Bhola NE Balko JM Dugger TC Kuba MG Sánchez V Sanders M. TGF-β inhibition enhances chemotherapy action against triple-negative breast cancer. J Clin Invest (2013) 123(3):1348–58. doi: 10.1172/JCI65416 PMC3582135 23391723

[157] Liu L Ye Y Zhu X. MMP-9 secreted by tumor associated macrophages promoted gastric cancer metastasis through a PI3K/AKT/Snail pathway. BioMed Pharmacother (2019) 117:109096. doi: 10.1016/j.biopha.2019.109096 31202170

[158] Shi X Gong L Liu Y Hou K Fan Y Li C. 4-phenylbutyric acid promotes migration of gastric cancer cells by histone deacetylase inhibition-mediated IL-8 upregulation. Epigenetics (2020) 15(6-7):632–45. doi: 10.1080/15592294.2019.1700032 PMC7574398 31814524

[159] Wang Y Sheng Q Spillman MA Behbakht K Gu H. Gab2 regulates the migratory behaviors and e-cadherin expression via activation of the PI3K pathway in ovarian cancer cells. Oncogene (2012) 31(20):2512–20. doi: 10.1038/onc.2011.435 PMC3262088 21996746

[160] Wöhrle FU Daly RJ Brummer T. Function, regulation and pathological roles of the Gab/DOS docking proteins. Cell Commun Signal (2009) 7:22. doi: 10.1186/1478-811X-7-22 19737390 PMC2747914

[161] Wang Y Xu RC Zhang XL Niu XL Qu Y Li LZ. Interleukin-8 secretion by ovarian cancer cells increases anchorage-independent growth, proliferation, angiogenic potential, adhesion and invasion. Cytokine (2012) 59(1):145–55. doi: 10.1016/j.cyto.2012.04.013 22579115

[162] Zarogoulidis P Katsikogianni F Tsiouda T Sakkas A Katsikogiannis N Zarogoulidis K. Interleukin-8 and interleukin-17 for cancer. Cancer Invest (2014) 32(5):197–205. doi: 10.3109/07357907.2014.898156 24669909

[163] Qi WQ Zhang Q Wang JB. CXCL8 is a potential biomarker for predicting disease progression in gastric carcinoma. Transl Cancer Res (2020) 9(2):1053–62. doi: 10.21037/tcr.2019.12.52 PMC8797801 35117450

[164] Haraguchi M Komuta K Akashi A Matsuzaki S Furui J Kanematsu T. Elevated IL-8 levels in the drainage vein of resectable dukes’ c colorectal cancer indicate high risk for developing hepatic metastasis. Oncol Rep (2002) 9(1):159–65. 11748475

[165] Li A Dubey S Varney ML Dave BJ Singh RK. IL-8 directly enhanced endothelial cell survival, proliferation, and matrix metalloproteinases production and regulated angiogenesis. J Immunol (2003) 170(6):3369–76. doi: 10.4049/jimmunol.170.6.3369 12626597

